# An analysis of operational efficiency and its influencing factors in traditional Chinese medicine hospitals in Shaanxi Province, China

**DOI:** 10.3389/fpubh.2025.1499134

**Published:** 2025-08-20

**Authors:** Jieming Zhang, Yan Li, Huiyang Qu, Penggang Chen

**Affiliations:** 1 Department of Hospital Information Network, The Second Affiliated Hospital of Xi’an Jiaotong University, Xi’an, China; 2 Department of Neurology, The Second Affiliated Hospital of Xi’an Jiaotong University, Xi’an, China

**Keywords:** bootstrap DEA model, Tobit model, hospital of traditional Chinese medicine, technical efficiency, operational efficiency, healthcare management

## Abstract

**Background:**

Over the past decade, traditional Chinese medicine (TCM) hospitals in China have experienced significant growth. However, their developmental status remains unclear, posing challenges to the formulation and optimization of relevant healthcare policies. This study aimed to assess the operational efficiency of TCM hospitals in Shaanxi Province and explore its influencing factors, thereby providing empirical evidence to support performance improvement.

**Methods:**

A total of 167 TCM hospitals in Shaanxi Province were included in this study. The Bootstrap Data Envelopment Analysis (Bootstrap-DEA) was utilized to calculate bias-corrected operational efficiency scores. Subsequently, Tobit regression analysis was used to identify significant determinants of efficiency, offering a comprehensive understanding of influencing factors.

**Results:**

After bias-correction, operational efficiency scores decreased across all hospitals. Tertiary hospitals had the highest mean operational efficiency score (0.457), followed by secondary hospitals (0.374), unclassified hospitals (0.354), and primary hospitals (0.329). Tobit regression indicated that the total number of visits, number of discharged patients, and bed occupancy rate positively influenced efficiency scores (*p* < 0.05), whereas number of employees and average length of stay had a negative impact (*p* < 0.05).

**Conclusion:**

The development of TCM hospitals in Shaanxi Province remains uneven, and overall operational efficiency is suboptimal. Enhancing efficiency requires targeted strategies, such as optimizing staffing levels, reducing the average length of stay, and improving bed utilization. These findings offer practical insights for policymakers to support the sustainable development of TCM hospitals.

## Introduction

Traditional Chinese Medicine (TCM), representing the accumulated wisdom of thousands of years, remains a cornerstone of Chinese cultural heritage. Its effectiveness has been repeatedly demonstrated during major public health crises such as the SARS and COVID-19 pandemics (1–3), reinforcing its role in global healthcare and stimulating growing international interest in TCM practices. As the primary institutions responsible for delivering TCM services in disease prevention, treatment, and health promotion, TCM hospitals play a crucial role in advancing this field. In response, the Chinese government has introduced a series of policy initiatives to promote TCM development. Key policy documents including the Outline of the Strategic Plan for the Development of Traditional Chinese Medicine (2016–2030) (4), the Law of the People’s Republic of China on Traditional Chinese Medicine (enacted 2017) (5), and the 14th Five-Year Plan for the Development of Traditional Chinese Medicine (6–8) have integrated TCM into national health strategy, aiming to establish a high-quality TCM service system by 2025. Consequently, TCM hospitals have expanded rapidly over the past decade. However, this growth raises critical questions: Are these hospitals operating efficiently? What factors contribute to performance disparities? Addressing these questions is essential to aligning TCM development with national strategic objectives.

Data Envelopment Analysis (DEA) and its Bootstrap extension have become widely adopted methods for evaluating hospital efficiency (9–14). While prior studies have examined the efficiency of TCM hospitals in regions such as Hunan and Zhejiang (15), most have relied solely on traditional DEA models, without correcting for statistical bias using the Bootstrap approach. This limitation may result in overestimated efficiency scores, as recent research has emphasized the importance of bias correction in efficiency evaluation (16, 17).

Moreover, existing research often neglects regional heterogeneity in TCM development. Shaanxi Province, a core region in northwest China with a deep-rooted TCM tradition, faces distinct challenges in balancing service expansion with operational efficiency. Despite this, TCM hospitals in the province remain insufficiently studied. Few studies have systematically analyzed how factors like hospital grade, geographical location, and TCM service structure influence efficiency across different hospital tiers, resulting in a critical gap in understanding region-specific efficiency determinants.

This study aims to employ the Bootstrap-DEA model to rigorously assess the operational efficiency of TCM hospitals in Shaanxi Province by correcting the bias inherent in traditional DEA assessments. The Tobit model is further employed to identify key influencing factors and examine their differential effects across hospital tiers. By providing evidence-based insights into the operational status of TCM hospitals, this research supports targeted policy interventions aligned with national strategic objectives. By integrating rigorous methodological corrections with regional focus, the study seeks to improve the accuracy of efficiency evaluation and address the current gap in understanding TCM hospital performance in northwest China.

## Materials and methods

### Data sources

Data were sourced from the National Health Statistics Network Direct Reporting System (18), maintained by the National Health Commission. This system is designed to enhance evidence-based decision-making by improving the collection, analysis, and utilization of health information. We extracted 2022 data for 184 TCM hospitals in Shaanxi Province, including variables such as hospital grade, geographical location, number of employees, actual available bed days, total number of visits, number of discharged patients, bed occupancy rate, average length of stay, and proportion of revenue from TCM services. After excluding 17 hospitals that either lacked inpatient beds or had no bed utilization, the final sample included 167 hospitals (11 tertiary, 101 secondary, 24 primary, and 31 unclassified). Indicator definitions are provided in Table 1.

**Table 1:** Explanations of research indicators.

Primary indicators	Secondary indicators	Description
Hospital grade	Primary, secondary, or tertiary hospital	A classification of hospitals by the health administration department according to the hospital scale, service quality, etc.
Geographical location	Provincial capital city or non-provincial capital city	The geographical location in a provincial capital city or a non-provincial capital city
Resource allocation	Number of employees	Personnel who work in hospitals and are paid wages by their units
	Actual available bed days	Sum of number of opening beds in hospital at 12:00 pm every day during the year
	Ratio of employees to beds	Ratio of number of employees to available beds
Workload	Total number of visits	Total number of people involved in all diagnosis and treatment work
	Number of discharged patients	Number of discharged patients after hospitalization during the year
Work efficiency	Occupancy rate of hospital beds	Ratio of actual using bed days to actual available bed days during the year
	Average length of stay in hospital	Average number of inpatient bed days occupied per discharge
	Proportion of revenue from TCM services	Proportion of revenue from TCM services (excluding drugs, consumables, and inspection and testing revenue) to total medical revenue

### Bootstrap-DEA model

Input and output indicators were selected based on relevant literature (11–15, 19) and data availability. The input variables were “Number of employees” and “Actual available bed days.” The output variables were “Total number of visits,” “Number of discharged patients,” and “occupancy rate of hospital beds.” Technical efficiency scores, representing operational efficiency under the assumption of constant returns to scale (CRS), were calculated using an input-oriented DEA model. To address the bias inherent in traditional DEA estimates, Bootstrap-DEA (2000 replications) was employed. For classification purposes and subsequent analysis, bias-corrected TE scores were used to categorize hospital efficiency into four levels: excellent, good, general, and worse.

### Tobit model

To further explore the factors influencing hospital operational efficiency and given the truncated nature of the bias-corrected efficiency scores, we adopted Tobit model. The independent variables included “geographical location,” “number of employees,” “actual available bed days,” “ratio of employees to beds,” “total number of visits,” “number of discharged patients,” “occupancy rate of hospital beds,” “average length of stay in hospital,” and “proportion of revenue from TCM services.” Subgroup analyses were conducted by hospital grade (primary, secondary, tertiary, unclassified). To improve the interpretability of the regression coefficients, several variables were rescaled as follows: number of employees (divided by 1,000), actual available bed days (divided by 100,000), total number of visits (divided by 100,000), number of discharged patients (divided by 10,000), occupancy rate of hospital beds (divided by 10), and proportion of revenue from TCM services (divided by 10).

### Statistical analysis

The original data were initially processed using Microsoft Excel, and subsequent statistical analyses were conducted in R software (version 4.2.2). Descriptive statistics including mean, standard deviation (SD), minimum, maximum, and interquartile range (IQR) were calculated for the indicators included in the DEA model. The DEA and Bootstrap-DEA models were realized using “rDEA” R package, while the Tobit model was realized using “VGAM” R package.

## Results

### Descriptive statistics

After excluding 17 hospitals that either lacked inpatient beds or reported no bed utilization, a total of 167 out of 184 TCM hospitals in Shaanxi Province were included in the analysis. The sample comprised 11 tertiary hospitals, 101 secondary hospitals, 24 primary hospitals, and 31 unclassified hospitals, distributed across 10 prefecture-level cities and one demonstration zone. As shown in Table 2, substantial variation in capacity was observed across all indicators, even within the same hospital grade.

**Table 2:** The descriptive statistics of input and output indicators of TCM hospitals.

Indicators	Mean	SD	Minimum	Maximum	Interquartile range (IQR)
Overall TCM hospitals (*n* = 167)					
Input indicators					
Number of employees	277.40	393.76	7.00	2285.00	265.50
Actual available bed days	81899.07	108318.13	365.00	662603.00	84497.50
Output indicators					
Total number of visits	92786.57	167088.99	140.00	1068498.00	93555.50
Number of discharged patients	5814.60	9089.63	6.00	53828.00	6805.50
Occupancy rate of hospital beds (%)	57.99	27.32	0.25	119.86	39.09
Primary TCM hospitals (*n* = 24)					
Input indicators					
Number of employees	33.04	18.98	7.00	81.00	16.75
Actual available bed days	16280.79	13008.19	365.00	60590.00	11352.50
Output indicators					
Total number of visits	10963.92	10523.3	700.00	39661.00	13254.25
Number of discharged patients	1048.08	1535.98	6.00	7452.00	979.50
Occupancy rate of hospital beds (%)	52.28	31.71	0.25	100.64	46.21
Secondary TCM hospitals (*n* = 101)					
Input indicators					
Number of employees	268.44	181.19	43.00	1067.00	241.00
Actual available bed days	79877.28	57652.29	3240.00	292000.00	74460.00
Output indicators					
Total number of visits	82120.42	75396.71	914.00	452504.00	96562.00
Number of discharged patients	5569.85	5302.36	12.00	23818.00	7075.00
Occupancy rate of hospital beds (%)	58.20	26.24	0.42	119.86	39.81
Tertiary TCM hospitals (*n* = 11)					
Input indicators					
Number of employees	1433.91	660.61	467.00	2285.00	1115.50
Actual available bed days	389694.82	180504.12	161330.00	662603.00	298024.00
Output indicators					
Total number of visits	552609.00	361138.58	119629.00	1068498.00	579606.00
Number of discharged patients	29958.00	17355.57	8025.00	53828.00	28325.50
Occupancy rate of hospital beds (%)	82.92	11.23	72.82	107.63	13.37

### Results of DEA analysis

The technical efficiency scores and their bias-corrected counterparts obtained through Bootstrap-DEA are described in Table 3. After Bootstrap correction, the efficiency scores of all hospitals decreased, with a mean bias of 0.107. The mean efficiency score decreased from 0.476 before correction to 0.369 after bias adjustment. After bias adjustment, the highest efficiency score was observed in Hospital No. 158 (0.758), while the lowest was recorded in Hospital No. 41 (−0.683).

**Table 3:** Operational efficiency scores before and after bias-correction of TCM hospitals.

No.	Before bias-correction	After bias-correction	Bias	Lower bound	Upper bound	Ranking orders*
158	0.859	0.758	0.101	0.682	0.862	1
87	0.763	0.710	0.052	0.669	0.779	2
126	0.784	0.703	0.081	0.641	0.799	3
27	1.000	0.676	0.324	0.429	0.970	4
137	0.719	0.659	0.061	0.610	0.733	5
22	0.840	0.654	0.186	0.500	0.861	6
36	0.893	0.637	0.256	0.430	0.862	7
121	0.695	0.635	0.060	0.587	0.706	8
134	0.503	0.462	0.041	0.430	0.517	54
93	0.766	0.458	0.308	0.197	0.757	55
150	0.497	0.457	0.040	0.425	0.505	56
85	0.511	0.452	0.060	0.403	0.516	57
5	0.584	0.447	0.137	0.332	0.609	58
115	0.599	0.447	0.152	0.330	0.611	59
52	0.559	0.447	0.113	0.365	0.564	60
65	0.500	0.446	0.054	0.404	0.503	61
161	0.350	0.323	0.027	0.302	0.358	107
64	0.382	0.316	0.066	0.262	0.379	108
120	0.352	0.313	0.039	0.282	0.358	109
19	0.378	0.312	0.066	0.265	0.373	110
155	0.352	0.302	0.050	0.260	0.355	111
118	0.392	0.300	0.092	0.223	0.384	112
119	0.342	0.292	0.049	0.257	0.343	113
79	0.374	0.288	0.086	0.223	0.371	114
117	0.104	0.081	0.023	0.063	0.103	160
61	0.096	0.074	0.022	0.054	0.101	161
55	0.104	0.067	0.037	0.034	0.097	162
23	0.071	0.055	0.016	0.042	0.070	163
60	0.052	0.037	0.015	0.023	0.056	164
56	0.031	0.022	0.008	0.015	0.031	165
17	0.011	0.008	0.003	0.005	0.012	166
41	1.000	−0.683	1.683	−2.277	5.470	167
Mean	0.476	0.369	0.107	/	/	/

*: Ranking by the bias-corrected operational efficiency scores.

**Figure 1:**
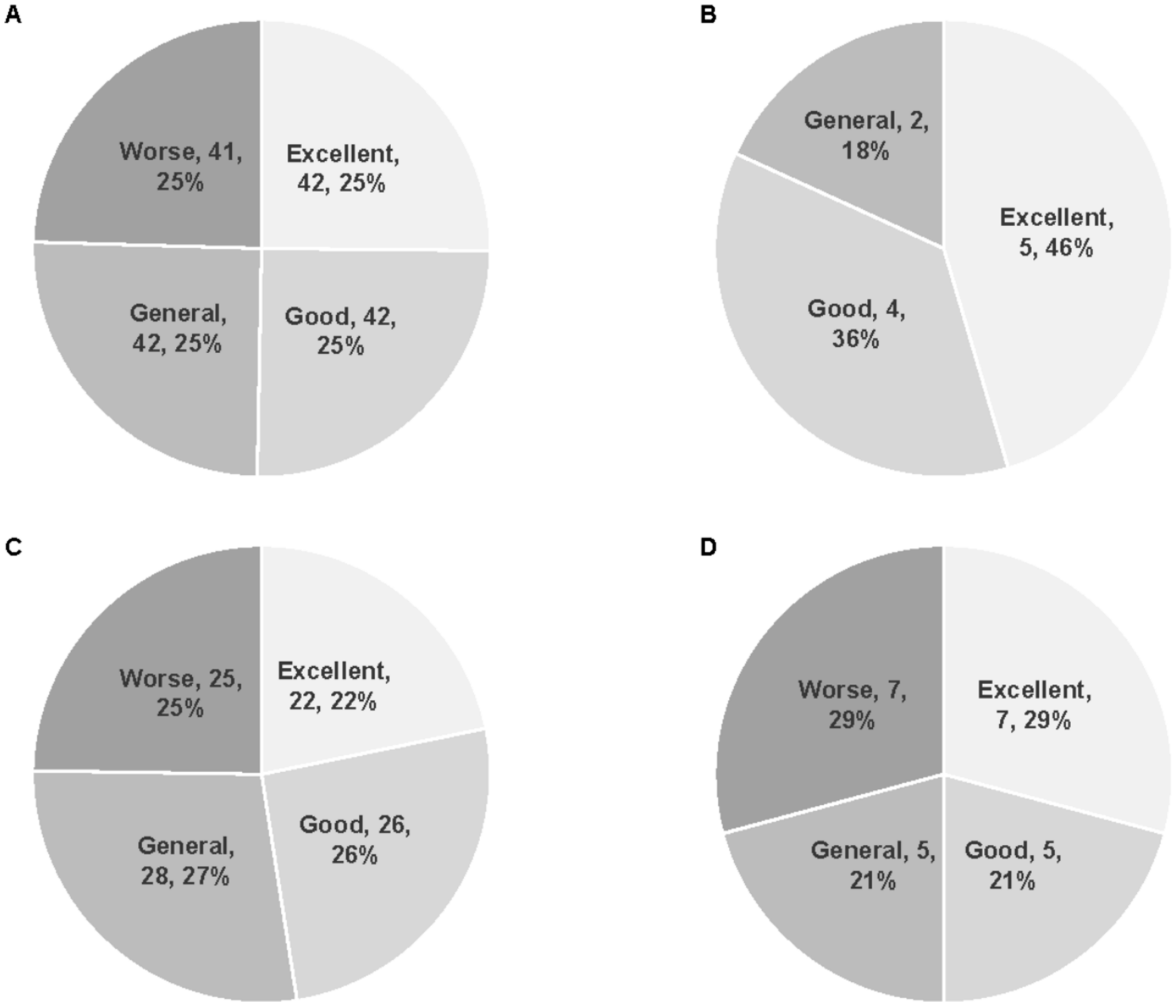
Distribution of hospital efficiency categories across different grades of TCM hospitals. **(A)** All 167 TCM hospitals. **(B)** Tertiary TCM hospitals (*n* = 11). **(C)** Secondary TCM hospitals (*n* = 101). **(D)** Primary TCM hospitals (*n* = 31).

The bias-corrected operational efficiency scores were highest among tertiary TCM hospitals (mean = 0.457), followed by secondary hospitals (0.374), unclassified hospitals (0.354), and primary hospitals (0.329). As described in Figure 1, tertiary hospitals had the largest proportion of institutions classified as “excellent” or “good.” In contrast, secondary hospitals exhibited a slightly lower proportion of high-efficiency institutions than primary hospitals.

### Results of Tobit analysis

After excluding one hospital (No. 41) with a negative bias-corrected operational efficiency score, a total of 166 TCM hospitals were included in the Tobit analysis. The results for both the overall sample and the subgroups by hospital grade are presented in Table 4.

**Table 4:** Results of Tobit model for overall TCM hospitals and different graded hospitals.

Primary variable	Secondary variable	Coefficient	Standard error	95% Confidence interval	*p*-value
Overall TCM hospitals (*n* = 166)					
Geographical location	Non-provincial capital city (reference = provincial capital city)	0.005	0.018	−0.029 to 0.04	0.764
Resource allocation	Number of employees	−0.544	0.102	−0.744 to −0.345	<0.001
	Actual available bed days	−0.009	0.038	−0.083 to 0.065	0.806
	Ratio of employees to beds	−0.001	0.008	−0.017 to 0.015	0.911
Workload	Total number of visits	0.094	0.017	0.061 to 0.127	<0.001
	Number of discharged patients	0.098	0.053	−0.005 to 0.201	0.063
Work efficiency	Occupancy rate of hospital beds	0.037	0.004	0.029 to 0.044	<0.001
	Average length of stay in hospital	−0.006	0.001	−0.008 to −0.004	<0.001
	Proportion of revenue from TCM services	−0.007	0.004	−0.016 to 0.001	0.092
Primary TCM hospitals (*n* = 23)					
Geographical location	Non-provincial capital city (reference = provincial capital city)	0.001	0.064	−0.125 to 0.128	0.982
Resource allocation	Number of employees	−6.283	3.371	−12.89 to 0.323	0.062
	Actual available bed days	0.471	0.728	−0.956 to 1.897	0.518
	Ratio of employees to beds	−0.051	0.101	−0.25 to 0.147	0.611
Workload	Total number of visits	1.231	0.376	0.494 to 1.968	0.001
	Number of discharged patients	−0.489	0.448	−1.368 to 0.39	0.276
Work efficiency	Occupancy rate of hospital beds	0.055	0.015	0.025 to 0.086	<0.001
	Average length of stay in hospital	−0.023	0.01	−0.043 to −0.004	0.021
	Proportion of revenue from TCM services	−0.015	0.016	−0.047 to 0.016	0.334
Secondary TCM hospitals (*n* = 101)					
Geographical location	Non-provincial capital city (reference = provincial capital city)	−0.019	0.019	−0.056 to 0.018	0.3059
Resource allocation	Number of employees	−0.476	0.108	−0.687 to −0.265	<0.001
	Actual available bed days	−0.069	0.046	−0.159 to 0.021	0.134
	Ratio of employees to beds	−0.005	0.007	−0.018 to 0.009	0.491
Workload	Total number of visits	0.147	0.017	0.113 to 0.181	<0.001
	Number of discharged patients	0.147	0.059	0.032 to 0.262	0.012
Work efficiency	Occupancy rate of hospital beds	0.024	0.005	0.015 to 0.033	<0.001
	Average length of stay in hospital	−0.003	0.001	−0.006 to 0	0.0349
	Proportion of revenue from TCM services	−0.012	0.006	−0.024 to −0.001	0.0366
Tertiary TCM hospitals (*n* = 11)					
Geographical location	Non-provincial capital city (reference = provincial capital city)	−0.233	0.005	−0.244 to −0.223	<0.001
Resource allocation	Number of employees	−0.099	0.013	−0.124 to −0.074	<0.001
	Actual available bed days	−0.23	0.007	−0.244 to −0.216	<0.001
	Ratio of employees to beds	−0.446	0.016	−0.477 to −0.415	<0.001
Workload	Total number of visits	−0.06	0.003	−0.066 to −0.054	<0.001
	Number of discharged patients	0.408	0.008	0.391 to 0.424	<0.001
Work efficiency	Occupancy rate of hospital beds	0.03	0.002	0.025 to 0.034	<0.001
	Average length of stay in hospital	0.004	0	0.004 to 0.005	<0.001
	Proportion of revenue from TCM services	−0.167	0.005	−0.176 to −0.157	<0.001
Unclassified TCM hospitals (*n* = 31)					
Geographical location	Non-provincial capital city (reference = provincial capital city)	−0.047	0.044	−0.134 to 0.039	0.282
Resource allocation	Number of employees	0.103	1.404	−2.648 to 2.854	0.942
	Actual available bed days	−0.043	0.482	−0.987 to 0.901	0.929
	Ratio of employees to beds	−0.075	0.079	−0.229 to 0.079	0.339
Workload	Total number of visits	0.305	0.145	0.02 to 0.59	0.036
	Number of discharged patients	−0.462	0.398	−1.242 to 0.317	0.245
Work efficiency	Occupancy rate of hospital beds	0.053	0.01	0.035 to 0.072	<0.001
	Average length of stay in hospital	−0.007	0.003	−0.013 to −0.001	0.021
	Proportion of revenue from TCM services	0.005	0.006	−0.007 to 0.018	0.413

At the 5% significance level, four variables were identified as significant determinants of bias-corrected operational efficiency in the overall sample: number of employees, total number of visits, bed occupancy rate, and average length of stay. Among these additional variables, total number of visits, number of discharged patients, and bed occupancy rate were positively associated with efficiency, while number of employees and average length of stay showed negative associations.

The results of the subgroup analyses varied by hospital grade. In primary TCM hospitals, three variables (total number of visits, bed occupancy rate, and average length of stay) significantly affected efficiency scores. In secondary hospitals, six variables (number of employees, total number of visits, number of discharged patients, bed occupancy rate, average length of stay, and proportion of revenue from TCM services) were significant. In tertiary hospitals, all nine independent variables were found to have a significant impact on operational efficiency.

## Discussion

### Heterogeneity in operational efficiency with bootstrap bias-correction

The overall operational efficiency of TCM hospitals in Shaanxi Province was relatively low, with a bias-corrected average efficiency score of 0.369. This is substantially lower than previously reported values for public TCM hospitals in Hunan Province (0.828) and TCM-related hospitals in Zhejiang Province (0.982) (15). Unlike these earlier studies, which employed the traditional DEA-BCC model without bias correction, our study applied the Bootstrap-DEA method to adjust for estimation bias. After correction, the average efficiency score in Shaanxi decreased from 0.476 to 0.369, indicating that the traditional DEA model may overestimate hospital efficiency. The application of Bootstrap bias correction provides a more accurate assessment by mitigating such overestimation and better capturing the heterogeneity of efficiency levels. Compared with national-level data, the operational efficiency of TCM hospitals in Shaanxi Province also appears slightly below average. In 2021, the national average comprehensive efficiency score for TCM hospitals was 0.852, and a declining trend in service efficiency was observed nationwide from 2017 to 2021. This suggests that the efficiency challenges in Shaanxi are not only region-specific but also part of a broader, systemic issue (19). Moreover, when compared with general Western medicine hospitals, the operational efficiency of TCM hospitals remains considerably lower. A nationwide study reported an average hospital efficiency score of 0.930, which far exceeds the levels observed in our study (20).

From the perspective of individual differences, after bias-correction, the highest operational efficiency score was 0.758 (Hospital No. 158), and the lowest is −0.683 (Hospital No. 41), yielding a range of 1.441. This wide dispersion reflects substantial variability in the capacity of hospitals to convert resources into services. Such heterogeneity may be attributed to differences in put structure and output quality. High-performing hospitals (e.g., Hospital No. 158) may achieve superior efficiency through optimal allocation of human and bed resources, whereas low-performing hospitals (e.g., Hospital No. 41) may experience inefficiencies due to resource redundancy or insufficient service output. These findings suggest that improving operational efficiency requires tailored interventions that address hospital-specific deficiencies. Interestingly, this pattern of heterogeneity also echoes the “high inelasticity of labor supply and demand to wages” observed in the UK healthcare system (21), highlighting the rigidity in resource reallocation. In our study, even within hospitals of the same classification level, the maximum number of employees was nearly 25 times greater than the minimum. This rigidity may stem from the high specificity of medical human capital and the substantial adjustment costs associated with healthcare workforce restructuring.

### Impact of hospital type on operational efficiency

Hospital grade represents a crucial source of heterogeneity in operational efficiency. After bias-correction, tertiary TCM hospitals exhibited the highest average efficiency score (0.457), followed by secondary (0.374), unclassified (0.354), and primary hospitals (0.329), revealing a clear efficiency gradient across hospital grades. This trend is consistent with findings from public TCM hospitals in Hunan Province, but differs from results reported in Zhejiang Province, where TCM-affiliated hospitals showed a different efficiency pattern (15). In terms of efficiency distribution, the proportion of tertiary hospitals classified as “excellent” or “good” was significantly higher than those secondary and primary hospitals (Figure 1), while primary hospitals had the largest proportion in the “worse” grade. These results suggest a positive association between hospital grade and operational efficiency. However, a study from Zhejiang Province found that the secondary public TCM hospitals outperformed tertiary ones in terms of efficiency, indicating that regional differences in hospital development stage, resource allocation strategies, and management capacity may influence the relationship between hospital type and efficiency (22).

It is noteworthy that 42.9% of tertiary hospitals operate under decreasing returns to scale, suggesting the presence of excessive resource allocation. This finding is consistent with an Australian study, which reported that a bed occupancy rate exceeding 85% will significantly reduce service quality (23). Hierarchical differences among TCM hospitals are also reflected in the output efficiency of TCM-characteristic services. In secondary hospitals, a negative correlation was observed between the proportion of revenue derived from TCM services and operational efficiency (*p* = 0.046), highlighting differences in how the value of TCM services is realized across hospital grades.

### Impact of geographical location on operational efficiency

The impact of geographical location on operational efficiency exhibits hierarchical dependence. Results from the Tobit model indicate that geographical location (provincial capital cities vs. non-provincial capital cities) has a statistically significant effect on operational efficiency only among tertiary hospitals (*p* < 0.001). Specifically, tertiary hospitals located in non-provincial capital cities show significantly lower efficiency compared to those situated in provincial capitals. In contrast, the effect of geographical location was not statistically significant for primary, secondary, and unclassified hospitals (*p* > 0.05). This discrepancy is attributed to differences in healthcare-seeking behavior. Tertiary hospitals are typically responsible for treating complex and severe conditions, and patients tend to prioritize technical competence over geographic proximity. Tertiary hospitals in provincial capitals, benefiting from preferential policy support, have developed stronger technical capacity and brand reputation, creating a “siphon effect” that attracts patients from surrounding areas (24). This inflow of patients increases service volume and contributes to improved efficiency. By contrast, primary-level hospitals mainly provide basic medical services, where patients are more concerned with accessibility. As a result, the influence of geographical location on resource acquisition and service output is less pronounced at the primary care level.

This finding is consistent with nationwide research, reflecting the persistent issue of regional disparities in the allocation of medical resources in China (20). Such differences align with a well-documented structural problem in the Chinese public health system, namely the concentration of healthcare resources in high-level institutions (25). Previous studies have demonstrated that regional GDP, resident population, and urbanization level significantly affect the efficiency of medical resource allocation (17, 26). Another study specifically highlighted marked differences in the efficiency of TCM hospitals across regions, indicating a clear imbalance in their development (19). From a regional perspective, the average operational efficiency of TCM hospitals was highest in the Guanzhong region (0.397), followed by the Northern Shaanxi region (0.345), and lowest in the Southern Shaanxi region (0.328). This gradient closely mirrors the level of regional economic development, underscoring the influence of regional economic conditions on hospital efficiency.

### Impact of TCM services on operational efficiency

This study found that the proportion of revenue derived from TCM services had a statistically significant negative impact on the operational efficiency of secondary and tertiary hospitals (*p* < 0.05), but no significant effect on primary hospitals (*p* > 0.05). These results reflect deficiencies in the value realization mechanism of TCM services (15). From a health pricing policy perspective, one possible explanation is that the pricing of TCM service items does not fully capture the technical value they provide, leading to a disproportionate income share that ultimately reduces overall efficiency (15). These findings are consistent with those of a national study on the allocation efficiency of TCM medical resources, which also highlighted the need to improve the cost compensation mechanism for TCM services (16). Moreover, a separate study on TCM hospital performance emphasized the importance of focusing on high-quality development, optimizing the external environment, and promoting coordinated regional development in order to enhance efficiency (27).

### Analysis of the pathways of influencing factors on operational efficiency

The Tobit model reveals multi-dimensional pathways through which various factors influence hospital operational efficiency, with the effects showing hierarchical variation. From the perspective of shared pathways, the total number of diagnoses and treatments, the number of discharges, and bed utilization rate all exert a significant positive impact on hospital efficiency (*p* < 0.05). These factors enhance efficiency by expanding service output and increasing the intensity of resource utilization. For instance, the bed utilization rate reflects the actual conversion efficiency of bed resources and serves as a key indicator of input–output alignment. In contrast, the number of employees and average length of stay have a significant negative impact on efficiency (*p* < 0.05), suggesting that excessive labor input or prolonged hospitalization contributes to resource waste and lower conversion efficiency (28). This finding aligns with conclusions drawn from UK studies, which indicate that the elasticity of human input and output is low (21), supporting the effectiveness of strategies such as staff optimization and shortening length of stay. However, attention must be paid to the threshold effects of certain variables. For example, when the bed occupancy rate exceeds 85%, it may result in adverse consequences such as increased risk of nosocomial infections and emergency department overcrowding (23). This finding provides a critical warning for bed management practices in TCM hospitals. It is suggested to increase investment in medical informationization and develop a dynamic bed adjustment platform, which can promote the improvement of bed utilization rate.

From the perspective of hierarchical differentiation, tailored strategies are needed to enhance operational efficiency across hospital levels. Tertiary hospitals should leverage their geographical advantages and economies of scale, while controlling the expansion of bed capacity within reasonable limits. Efforts should focus on improving both the quantity and quality of services. Additionally, they are advised to adjust the pricing of TCM services to better reflect their technical value and to strengthen the cost compensation mechanism (29). Secondary hospitals should aim to balance the proportion of TCM services with appropriate pricing mechanisms. Operational efficiency can be improved by reengineering clinical workflows to shorten the average length of stay and increase bed occupancy. Furthermore, optimizing human resource allocation and reducing redundant personnel will help release additional efficiency potential (16, 30). Primary hospitals should prioritize the refined management of basic resources, particularly bed and human resources, to improve their utilization efficiency. At the same time, they should consider moderate expansion of service capacity based on local demand, thereby enhancing their ability to deliver primary care services (31).

### Policy recommendations: pathways for promoting the high-quality development of TCM hospitals

Given the observed negative correlation between the number of employees and operational efficiency, as well as the low elasticity of the medical labor market, it is essential to strengthen investment in TCM talent development. Drawing on the UK’s experience (21), expanding training programs can help alleviate structural shortages in the TCM workforce. In light of the significant negative impact of TCM service income proportion on the efficiency of secondary and tertiary hospitals, efforts should be made to reform the pricing and cost compensation mechanisms for TCM services to better reflect their technical and service value (15).

Furthermore, a tiered development strategy is recommended. Tertiary TCM hospitals in provincial capital cities should be strengthened as regional leaders by establishing comprehensive regional TCM medical centers that integrate clinical care, teaching, research, preventive healthcare, and rehabilitation services. Secondary TCM hospitals should be positioned as regional backbones with a focus on improving their capacity to manage common and frequently occurring diseases through TCM diagnosis and treatment. Primary TCM hospitals should focus on reinforcing basic medical and public health services to improve the accessibility, affordability, and equity of TCM at the grassroots level.

### Limitations and future studies

Although we have conducted extensive empirical research on data, there are inevitably some research limitations, such as: 1. The data was cross-sectional and cannot capture the dynamic changes in efficiency; 2. Not included in policy variables such as medical insurance payment methods; 3. Micro mechanisms such as financial incentives have not been directly quantified. 4. The impact of personnel structure differences on hospital operational efficiency has not been fully considered, and it is hoped that data on physician structure can be included in future research. In response to these limitations, future research will adopt panel data models, incorporate policy variables, and design a “salary efficiency ratio” indicator to conduct in-depth analysis of incentive mechanisms.

## Conclusion

To promote the high-quality development of TCM hospitals, this study highlights the importance of advancing the standardization of information systems to enable interoperability and data sharing across internal hospital platforms. Additionally, the innovation of the “Internet + TCM” service models, such as telemedicine and online consultations, should be actively pursued to enhance service accessibility and operational efficiency.

Based on the empirical findings, several policy directions are proposed, including optimizing the allocation of regional TCM medical resources, enhancing internal management and operational efficiency, refining the pricing and compensation mechanisms for TCM services, promoting tier-specific and differentiated development strategies across hospital levels, and accelerating the digital transformation of TCM institutions. These strategies collectively aim to guide TCM hospitals toward sustainable and high-quality development, and to fully leverage the unique advantages and public health value of traditional Chinese medicine in the evolving healthcare landscape.

## Data Availability

The raw data supporting the conclusions of this article will be made available by the authors, without undue reservation.
