# Correction: An analysis of operational efficiency and its influencing factors in traditional Chinese medicine hospitals in Shaanxi Province, China

**DOI:** 10.3389/fpubh.2025.1759504

**Published:** 2026-01-09

**Authors:** Jieming Zhang, Yan Li, Huiyang Qu, Penggang Chen

**Affiliations:** 1 Department of Hospital Information Network, The Second Affiliated Hospital of Xi'an Jiaotong University, Xi'an, China; 2 Department of Neurology, The Second Affiliated Hospital of Xi'an Jiaotong University, Xi'an, China

**Keywords:** bootstrap DEA model, Tobit model, hospital of traditional Chinese medicine, technical efficiency, operational efficiency, healthcare management

Affiliation Department of Neurology, The Second Affiliated Hospital of Xi'an Jiaotong University, Xi'an, China was omitted for authors Jieming Zhang, Yan Li and Huiyang Qu. This affiliation has now been added for authors Jieming Zhang, Yan Li and Huiyang Qu.

Author Jieming Zhang was erroneously assigned as corresponding author. The correct corresponding author is Penggang Chen.

The original version of this article has been updated.

